# Explainable Artificial Intelligence (xAI) for 5-HT_2A_ Receptor Binding Affinity of New Psychoactive Substances

**DOI:** 10.3390/molecules31111888

**Published:** 2026-06-01

**Authors:** Verena Schöning, Katharina Elisabeth Grafinger, Daniel Pasin, Christophe P. Stove, Wolfgang Weinmann, Felix Hammann

**Affiliations:** 1 Clinical Pharmacology & Toxicology, Department of Internal Medicine, University Hospital Bern, 3010 Bern, Switzerland; 2 Institute of Forensic Medicine Bern, Forensic Toxicology and Chemistry, University of Bern, 3008 Bern, Switzerland; 3 Hyperion Data, Griffith, NSW 2680, Australia; 4 Laboratory of Toxicology, Department of Bioanalysis, Faculty of Pharmaceutical Sciences, Ghent University, 9000 Ghent, Belgium

**Keywords:** 5-HT_2A_, machine learning, binding affinity, explainable artificial intelligence, NPS, classification model

## Abstract

New psychoactive substances (NPS) are a heterogeneous group of recreational drugs that mimic the actions and psychoactive effects of existing pharmaceutical products or recreational drugs. As NPS can be highly potent, even exceeding their template compound’s potency, there are frequent reports of non-fatal and fatal intoxications. One principal target for hallucinogenic and psychedelic drugs, including NPS, is the 5-hydroxytryptamine receptor 2A (5-HT_2A_). Since NPS are designed to evade legal restrictions, this drug market is quickly evolving, and researchers are playing catch-up, investigating these novel compounds for their toxicological and pharmacological properties. Receptor binding affinity (Ki) is an important property describing ligand–receptor interactions and is a prerequisite for receptor activation. Competitive in vitro assays can be used to assess Ki; this is a resource-intensive process. We used publicly available Ki data for the 5-HT_2A_, calculated molecular descriptors and fingerprints, and trained five classification machine learning models. The predictive performance of the models had precisions and recalls up to 93% and 92%, respectively. We used explainable artificial intelligence, i.e., SHAP values and similarity maps, for model interpretation. The results are in line with previous experiments and support its suitability to predict the binding affinities of possible 5-HT_2A_ ligands.

## 1. Introduction

New psychoactive substances (NPS) are a heterogeneous group of recreational drugs, also known as designer drugs, synthetic drugs, herbal drugs, bath salts, research chemicals, or legal highs [1,2]. They are either analogs or newly synthesized substances that mimic the actions and psychoactive effects of existing pharmaceutical products or recreational drugs [1,3]. The manufacturers of NPS circumvent the stringent national and international regulations by exploiting loopholes within drug regulation, speed of development, and lag in the legislative process [4]. Especially for the latter, proof of harmfulness is often required, which in turn results in lengthy and costly studies. As NPS can be highly potent, with their potency even exceeding that of the template prototypical drug, non-fatal and fatal intoxications are frequently reported [5,6,7]. NPS can be categorized according to their chemical structures or function into different classes, such as synthetic cannabinoid receptor agonists, cathinones, phenethylamines, piperazines, tryptamines, and novel synthetic opioids [8]. Due to the constant and fast-evolving NPS drug market, researchers are playing catch-up, investigating these novel compounds for their toxicological and pharmacological properties. In this context, the receptor binding affinity is an important characteristic, as ligand binding to a receptor is a prerequisite for receptor activation [9]. However, receptor affinity is not equivalent to the toxicity of a compound. Receptor affinity relates to the strength of binding between a drug and its receptor, indicating how tightly the drug associates with the receptor. In contrast, efficacy (or intrinsic activity) is the drug’s ability to activate the receptor once it has bound, producing the desired biological response. The binding affinity of a compound is commonly tested using competitive radioligand assays, in which a radio-labeled high-affinity ligand competes with the test compound for binding to a given receptor [10]. In the past decade, this method has been applied for a series of NPS such as psychedelic tryptamines [11] and phenethylamines [12]. However, these assays are time-consuming and expensive. Therefore, alternative methods utilizing machine learning (ML) are increasingly gaining interest.

Of the 14 known types of serotonin receptors, which bind to the neurotransmitter serotonin (5-HT), the 5-hydroxytryptamine receptor 2A (5-HT_2A_) is the principal target for hallucinogenic and psychedelic drugs, including some classes of NPS, such as the phenethylamines and tryptamines [13]. Within the central nervous system (CNS), the 5-HT_2A_ is highly expressed in all layers of the cerebral cortex, with layer 5 having the highest concentration [14]. However, the 5-HT_2A_ is also found in other areas such as the intestinal tissue, platelets, and endothelial cells [14]. In the CNS, this receptor plays a central role in the regulation of cortical function and cognition, with neuropsychological effects including hallucinations, altered states of consciousness, cognition, mood, and perception [15]. Overstimulation of the serotonin system might lead to serotonin syndrome, a potentially fatal condition with symptoms such as tachycardia, hypertension, hyperthermia, and convulsions [16]. In previous research, three chemical classes were identified as ligands of 5-HT_2A_: tryptamines, ergolines, and phenethylamines [17].

Previous studies have already trained classification and regression machine learning models to predict the binding affinity of substances to 5-HT_2A_. Warszycki et al. (2017) [18] used a dataset of 2060 active and 1081 inactive 5-HT_2A_ ligands to identify relevant structural differences defining the affinity. However, active substances were defined as having a Ki ≤ 100 nM, which is not necessarily suitable for high affinity NPS. Furthermore, the authors provided no information regarding model performance. In another study, Łapińska et al. (2024) [19] trained two classification models, using Mordred molecular descriptors. The first model was designed to predict whether a ligand is active (pKi ≥ 7, i.e., 100 nM) for any of the serotonin receptors in the study. The second one was a multi-class classification model to distinguish which ligand binds to which serotonin receptor. However, the training data set was highly imbalanced, and the general approach to transform a multi-class classification problem into a regression problem might have adversely affected the reliability. Other attempts were made to develop regression models to predict binding affinity. Floresta et al. (2021) [20] used a relatively small dataset of only 375 ligands with experimentally determined Ki values for human 5-HT_2A_ to develop regression ML and field-based quantitative structure–activity relationship (QSAR) models. The endpoint was the negative decimal logarithm of the affinity (pKi), and the extended electron distribution (XED) was used to calculate the field points for each molecule. The squared correlation coefficient (R^2^) value on the test set was only 0.73, which means that 27% of the variability in the dependent variable is unexplained by the model. In a further study, Łapińska et al. (2024) [21] used 6926 substances with known pKi values for 5-HT_2A_. They trained a regression eXtreme gradient boosting (XGB) model using Mordred molecular descriptors.

However, the aforementioned approaches are not ideally suited for hallucinogenic NPS, as those potent substances most often have high binding affinities, i.e., a low Ki value, to their target receptor. Therefore, we developed different classification machine learning models with a special focus on a high-affinity class with binding affinities in the low nanomolar range. Furthermore, we used feature- and structure-based explainable artificial intelligence (xAI) paradigms to gain further insight into the model predictions.

## 2. Results

### 2.1. Dataset

The compiled dataset consisted of 9470 instances of 7436 (79%) unique substances. A competitive radioligand assay was used in 8687 (91%) instances, with [^3^H]ketanserin being the most common radioligand (76%), followed by [^125^I]DOI (1-(4-(125I)iodanyl-2,5-dimethoxyphenyl)propan-2-amine, 8%) and [^3^H]DOB (2,5-Dimethoxy-4-bromoamphetamine, 1%). The most common receptor species were human (63%) and rat (32%), and the most common cell membranes were from HEK (human embryonic kidney) cell lines (27%), various brain tissues (24%) and CHO (Chinese hamster ovaries) cell lines (21%). The mean pKi value (negative logarithm of the Ki) was 7.16 ± 1.32. We subsequently only used results that were obtained using a competitive radioligand assay.

### 2.2. Outcome Engineering

After comparing the different outcome definitions, we decided to use a multi-class problem with the following class definitions: (i) high-affinity (Ki < 10 nM), (ii) low-affinity (1000 nM > Ki ≥ 100 nM), and (iii) no-affinity (Ki > 3000 nM). Substances where the outcome was defined as “unspecified” or “inconclusive” in PubChem are also part of the no-affinity class. This resulted in a reduced dataset with 5027 instances. The share of substances in each class was relatively balanced, with approximately 35%, 33% and 32% in the high-affinity, low-affinity and no-affinity classes, respectively (Supplemental Information, Figure S1).

### 2.3. Comparison of Machine Learning Algorithms and Predictor Data Sets

We trained five different ML algorithms, i.e., eXtreme gradient boosting (XGB), Random Forest (RF), Support Vector Machines (SVM), Multi-layer perceptron (MLP), and logistic regression (LR), with four different predictor datasets, i.e., molecular descriptors, MACCS (Molecular ACCess System), ECFP (extended connectivity fingerprints, Morgan fingerprints with radius of 3), and KRFP (Klekota Roth fingerprints) (Table 1). The best-performing predictor–algorithm combinations on the test dataset were ECFP with XGB and RF, with recall and precision scores of 91–93%. Molecular descriptors with XGB, KRFP with RF and ECFP with MLP also showed a high predictive performance (recall and precision scores on the test dataset 89–91%). The confusion matrices for all models on the test dataset are provided in the Supplemental Information (Figures S2–S6).

### 2.4. Y-Randomization

We validated our model by performing a y-randomization using XGB. On the training data set, the precision, recall, and F1 score ranged from 70% (with KRFP) to 98% (molecular descriptors) (Supplemental Information, Table S1). However, in the test data set, the metrics were between 43% and 49%.

### 2.5. SHAP Values

**Figure 1:**
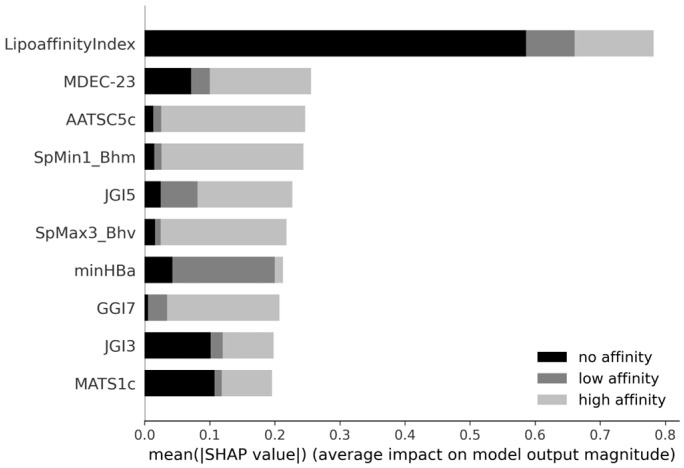
Summary plot for the overall 10 most important molecular descriptors (explanation of molecular descriptor abbreviations see Supplemental Information, Table S2).

**Figure 2:**
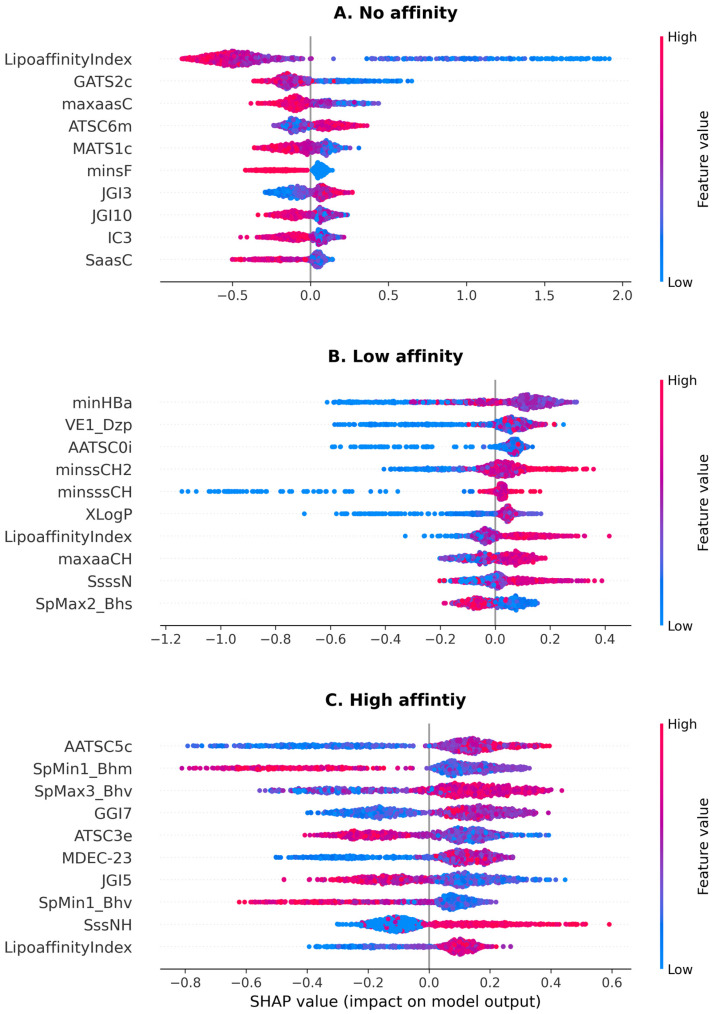
Bee-swarm plots for the ten most important features per class (explanation of molecular descriptor abbreviations see Supplemental Information, Table S2).

The ten most important molecular descriptors and their mean overall SHAP value are presented in Figure 1, and the bee-swarm plots for each class are provided in Figure 2. Further explanation of the mentioned single molecular descriptors is provided in the Supplemental Information, Table S2.

The most important molecular descriptor to determine the binding affinity of a substance to 5-HT_2A_ is the lipoaffinity index, i.e., the affinity for lipophilic binding sites. Lower lipoaffinity values are more predictive for the no-affinity class, for which this descriptor is also the most important one. The second most important molecular descriptor is MDEC-23, the molecular distance between all secondary and tertiary carbons, which is mainly predictive of the high-affinity class. This descriptor basically reflects the compactness (or branching) of a molecule for distances of two and three. This quality can influence lipophilicity, steric hindrance, and the potential for target engagement, i.e., the biological activity of a molecule and its capability to reach its target across tissue barriers.

In summary, four classes of molecular descriptors are mainly represented in the overall and class-specific top-10 molecular descriptors: (i) Atom-type electrotopological state (LipoaffinityIndex, maxaaCH, maxaasC, minaaaC, minHBa, minHBint5, minsF, minssCH, minssCH2, SaasC, SssNH, SsssN), (ii) Autocorrelation (AATSC0i, AATSC5c, ATSC3e, ATSC6m, GATS2c, GATS5i, MATS1c), (iii) Burden modified eigenvalues (SpMax2_Bhs, SpMax3_Bhv, pMin1, Bhm, SpMin1_Bhv), and (iv) Topological charge (GGI5, GGI7, JGI10, JGI3, JGI5) (Supplemental Information, Table S2).

In brief, these descriptors capture the structural, electronic, and physicochemical features of molecules that influence blood–brain barrier (BBB) penetration and CNS activity [22]. Atom-type electrotopological indices reflect lipophilicity, hydrogen bonding, and atom-specific electronic environments. These are crucial for balancing permeability with receptor binding. Autocorrelation, Burden eigenvalues, and Topological charge indices further quantify how electronic distribution and molecular topology affect transport, distribution, and interaction with CNS targets. Most of the latter group are strictly mathematical representations of the molecular connectivity matrices (i.e., tables that map the quantity or quality of connections every atom engages in). Though abstract, they have proven useful in providing statistical learners with information on complex and highly dimensional properties of molecular structures [22].

### 2.6. Similarity Maps

**Figure 3:**
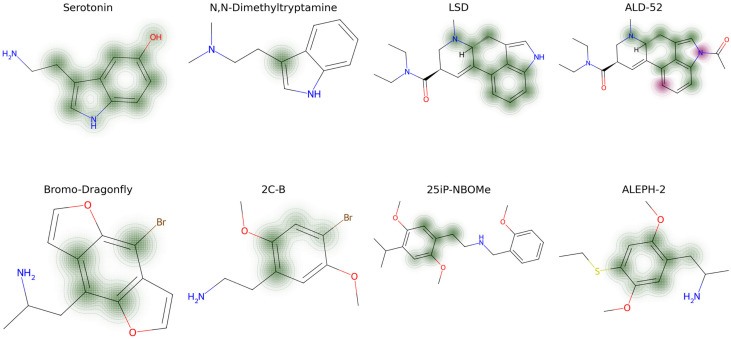
Similarity maps of eight selected substances. Green indicates a positive influence on the prediction outcome, i.e., removal of the atom would decrease the prediction probability, whereas pink indicates a negative influence.

We created similarity maps for eight selected substances (Figure 3). Firstly, we chose serotonin, a tryptamine derivative, as this is the endogenous ligand for 5-HT_2A_. The indole ring, the 5-hydroxyl substituent and the first carbon of the secondary amine substituent are considered relevant for the classification as a high-affinity substance by the XGB model. N,N-dimethyltryptamine (DMT) is also a tryptamine derivative with a tertiary amine group. The carbon atom at the indole ring with the tertiary amine group contributes to the classification as a low-affinity substance. The presence of such an atom increases polarity and capacity for hydrogen bonding, making it less prone to permeate the BBB. The ergolin derivative LSD (lysergic acid diethylamide) is a semisynthetic hallucinogenic drug derived from the ergot fungus. The most relevant substructures for the prediction are the benzene ring and partly the cyclohexene and tetrahydropyridine, both of which define its binding potential. ALD-52, also known as 1-acetyl-LSD, was classified as low-affinity. The nitrogen with the ketone group negatively influenced the prediction, as well as one carbon of the benzene ring. Otherwise, the influences of the different atoms are comparable between LSD and ALD-52. We furthermore chose four different hallucinogenic NPS, all phenethylamine derivatives, i.e., Bromo-Dragonfly, 2C-B, 25iP-NBOMe and ALEPH-2. Here, the benzene ring, especially if the carbon carries an amine or methoxy substituent, is identified as important for the prediction in the high-affinity class. Most likely, this is owing to the rigidity of benzene rings. The addition of substituents may improve hydrogen bonding or electronic modulation of the aromatic ring, making them highly binding ligands.

## 3. Discussion

In this study, we compiled a dataset of substances with experimentally measured 5-HT_2A_ binding affinity. For each substance, we calculated four different predictor sets. We tested different class definitions for our multi-class problem. We evaluated different approaches for splitting the training and test datasets to obtain optimal coverage of the applicability domain with the available substances. We compared the performance of five different ML algorithms. We validated our model using y-randomization. Lastly, we used SHAP values and similarity maps to explain the features’ contribution to the prediction.

All combinations of models and predictors showed robust (F1 score > 80%) to excellent (F1 score > 90%) performance. Therefore, all the used models and predictors are generally suitable for predicting the binding affinity class of a substance to 5-HT_2A_ within the applicability domain of the models.

Our validation using y-randomization showed that the predictor information in the training dataset is so dense that the algorithms could even learn and “predict” random outcomes. However, when applied to the test dataset, performance metrics dropped below 50%, indicating essentially random prediction. This outcome confirms that the y-randomization procedure was successful in ruling out chance correlations. In contrast, when using the actual outcomes, the models achieved high performance metrics on the test set, supporting the validity and robustness of the modeling process. Furthermore, in general, these results underscore the importance of constructing and evaluating a proper test dataset to reliably assess model performance.

The insights provided by xAI into the model classification process supported the validity of the predictions. With regard to the SHAP values of molecular descriptors, the lipoaffinity of a molecule seems to be a crucial feature to determine binding affinity for the XGB model. Here, especially low lipoaffinity values, i.e., low affinity to lipophilic binding sites, are associated with no-affinity class predictions. Like most serotonin receptors, 5-HT_2A_ belongs to the family of G protein-coupled receptors (GPCRs). All GPCRs are composed of a set of seven α-helices, which are embedded in the cell membrane, connected by three intracellular and three extracellular loops. In the largest class of GPCRs, class A, the ligand binds directly to the transmembrane domain, also referred to as the binding pocket [23]. The transmembrane domain has highly hydrophobic residues and thus forms a hydrophobic core [24]. Therefore, the importance of the lipoaffinity of a ligand aligns with biochemical and molecular observations. An earlier study found a highly significant linear correlation between binding affinity and lipophilicity of 5-HT_2A_ ligands [25]. Other studies also found that a feature describing lipophilicity (Wildman–Crippen clogP) was relevant for the prediction of serotonergic activity at 5-HT receptors in general [19,26].

In addition to SHAP values, we used similarity mapping to visualize the importance of single atoms for classification. In detail, we analyzed the influence of different atoms of two tryptamines, i.e., serotonin and DMT, on the model prediction. While DMT was in the low-affinity group, serotonin was classified as high-affinity, with the oxygen atom at the 5-position influencing the classification. These observations are in accordance with previous studies, where DMT was reported to have a Ki of 75–440 nM [27,28]. Furthermore, binding affinity to 5-HT_2A_ is generally increased by an oxygen atom at the 4- or 5-position [17].

This study has some limitations. A limitation of our approach is that the modeling strategy may restrict the applicability domain. This arises from our decision to focus on high affinity substances, such as hallucinogenic NPS, which led us to apply a strict cutoff for high affinity (<10 nM), thereby introducing gaps between affinity groups and excluding compounds accordingly. Also, some domain experts might argue that a Ki of 3000 nM does indicate no affinity. While this is true, for our study, focusing only on high affinity substances, we deemed this approach appropriate. Furthermore, even though the results of the similarity maps were in alignment with previous studies, some authors may argue that the coloration of atoms is not always robust [29].

Several recent studies investigated the importance of biased agonism in the signaling pathways. Those studies indicate that certain pathways mediate hallucinogenic effects [30,31,32]. While identifying the signal pathway that mediates the hallucinogenic effects of a xenobiotic is important, our primary aim in this study is to correctly classify compounds with potential toxic or lethal effects. In this context, our focus on the 5-HT_2A_ receptor affinity reflects its role in enabling receptor engagement at pharmacologically relevant concentrations. Such engagement is a prerequisite for downstream signaling effects that potentially lead to adverse serotonergic outcomes, including serotonin syndrome [33]. It is worth noting, however, that these effects cannot be inferred from affinity alone.

ECFPs are circular fingerprints that do not rely on predefined substructural fragments. Instead, they create bits that encode the local chemical environment around each atom up to a specified radius, thereby capturing detailed and diverse structural information, including substructures relevant to biological activity [34]. However, this flexibility can reduce interpretability, as the resulting representation may include overlapping, ‘colliding’ bits, i.e., different fragments could be converted into the same bit [35,36].

In conclusion, we successfully built various classification models with excellent predictive performance. We validated an external test dataset and y-randomization to validate the models. The use of xAI, i.e., SHAP values and similarity maps, provided further insight into the model prediction. Prospective experimental validation of our models is planned for future work.

## 4. Materials and Methods

### 4.1. Data Compilation

During September 2024, we downloaded binding affinity data for 5-HT_2A_ from PubChem (https://www.ncbi.nlm.nih.gov/pcassay/, accessed on 13 September 2024). We compared the information provided by PubChem with the original literature and corrected the entries if necessary. Specifically, we excluded prediction from machine learning algorithms, unclear results (e.g., contradictory information within the publication), wrong records (e.g., experiments not related to 5-HT_2A_) and records that were not part of the original research (citations from other publications). Research, which was referenced in publications but not part of the PubChem database, was also included. Furthermore, we additionally searched PubMed for further publications published between 2020 and 2025.

Chirality information was considered where available from the PubChem database. In particular, isomeric SMILES were used to retain stereochemical information during descriptor calculation. However, due to the large size of the dataset (>9000 compounds), it was not feasible to systematically verify stereochemical annotations against the primary literature sources. As a result, some compounds may lack explicit stereochemical information or include unresolved stereochemistry.

### 4.2. Pre-Processing

We standardized SMILES (Simplified Molecular Input Line Entry System) using the molvs package (version 0.1.1), a Python tool leveraging RDKit to validate and standardize molecular information, to be able to identify identical molecules, even when different sources provided the SMILES. We only kept the largest fragment of each substance to remove counter-ions and other adjuncts. We then removed duplicates from the dataset, only keeping the record with the highest binding affinity. If duplicated records included experiments with numeric Ki values and “unspecified” or “inconclusive” results, we retained the record with the lowest Ki value, i.e., the highest binding affinity.

We then calculated PaDEL molecular descriptors [37] (length = 1444), MACCS (length = 166) fingerprints, ECFP (extended connectivity fingerprints, Morgan fingerprints with radius of 3, length = 2048) [36], and KRFP (length = 4860) [38].

To reduce dimensionality of the datasets, we performed an unspecific (outcome-independent) feature selection. For the molecular descriptor dataset, we removed all columns with more than 10 non-available (NA) values (i.e., values that were not computed). We then removed all rows with NAs, resulting in a table with no NA values. Columns with low variability, defined as less than 10% unique values, were also removed. All but one highly correlated descriptor, with a threshold of 0.95, were also dropped. We chose the threshold based on experience. For all the fingerprint datasets, we only dropped columns with constant values (the same value for all rows). The final datasets (PaDEL, MACCS, ECFP, and KRFP) used in this study are available at https://github.com/cptbern/5HT2A_ML and at https://zenodo.org/records/19493235.

Lastly, we split the datasets into a training (80%) and a validation (20%) dataset. To maximize the applicability domain based on molecular features, we calculated Morgan fingerprints (radius of 2) of the substances and then used a specific picking strategy aimed at diversity of the dataset [39], while also stratifying for the outcome. This resulted in a split that maximized the diversity of the dataset with regard to molecular features and is overall independent of the outcome variable. This approach also reduces the risk of information leakage arising from structurally similar compounds within chemical series by ensuring separation in chemical space rather than relying on discrete scaffold definitions. Notably, given the high scaffold diversity of the dataset (~2300 scaffolds for ~5000 substances in the final datasets), scaffold-based splitting would lead to substantial fragmentation and limited statistical robustness, and was therefore not considered appropriate in this context.

### 4.3. Outcome Engineering

Due to the high variability within the experimental data, we decided to train a binned (n-ary) classification model. We optimized our class definition by (a) comparing different thresholds for the single classes, (b) introducing gaps between the classes, and (c) evaluating different approaches for dealing with unspecific and inconclusive data, as defined by PubChem, in the dataset.

### 4.4. Comparison of ML Algorithms and Predictor Data Sets

Using the four different calculated descriptors and fingerprint datasets (molecular descriptors, MACCS, ECFP, KRFP), we trained and compared five different machine learning algorithms (XGB, RF, SVM, MLP and LR). For the SVM, MLP and LR models, we standardized the molecular descriptors to a mean of zero and unit variance.

As the performances of MLP and SVM models were notably lower, we performed a 5-fold cross-validated grid search on the training dataset to optimize hyperparameters.

### 4.5. Y-Randomization

To ensure that the models pick up actual signals and not merely noise, we performed y-randomization [40] as an additional validation step. We used the optimal outcome definition and the best-performing model (XGB) from the previous experiments for y-randomization. We shuffled the outcome variable, keeping the instances per class equal. Then we trained the model and compared the performance on the training and test datasets.

### 4.6. Explainable Artificial Intelligence

Human trust in ML models and their predictions is often hampered by the complexity of the underlying algorithm and decision-making process, and fostering trust could aid in establishing these techniques in a legal and regulatory framework. Explainable artificial intelligence (xAI) uses different techniques to enhance the understanding of model predictions. It also confirms that predictions are not based on noise within the dataset. For this study, we decided to use two different approaches to explain the model prediction based on the type of predictors, i.e., molecular descriptors or molecular fingerprints.

Molecular descriptors are numerical, mostly continuous, and often engineered molecular features that describe the physicochemical properties of the molecule. A feature-based approach is often the most appropriate choice. We used SHAP (Shapley Additive exPlanations) to identify the most informative attributes for the prediction of the XGB model [41,42].

Molecular fingerprints are high-dimensional, sparse, binary vectors encoding the presence or absence of chemical structures. They are a computationally efficient method for handling and comparing chemical structures. However, especially when those fingerprints are used to train machine-learning models, the interpretation of the results can pose a challenge [41]. For fingerprints, SHAP values are not suitable since a single substructure carries little intuitive meaning. Therefore, we decided on a structure-based approach. We used similarity maps to allow interpretation of the predictions of the XGB model with ECFP [43]. Here, the importance of an atom is the predicted probability difference between the presence and the absence in the molecule. Those weights were then used to highlight the atoms in a topography-like map. Green is used in these maps to reflect a positive influence on the prediction outcome, i.e., removal of the atom would decrease the probability. Pink highlights atoms with a negative impact on the prediction outcome [43].

### 4.7. Software

The study was conducted using Python Programming Language, version 3.13.0 [44]. We used RDKit, version 2024.09, to calculate ECFP descriptors and create similarity maps. We calculated PaDEL molecular descriptors [37], MACCS, and KRFP using padelpy, version 0.1.16. Machine learning, including the RF, SVM, and LR models, was conducted in scikit learn version 1.5.2 [45]. The XGB model was created using xgboost, version 2.1.3.

## Figures and Tables

**Table 1:** Comparison of the performance of different machine learning algorithms and predictor data sets.

		Train			Test		
Model	Predictors ^1^	F1	Precision	Recall	F1	Precision	Recall
XGB	MolDesc	1.00	1.00	1.00	0.89	0.90	0.89
	ECFP	0.98	0.98	0.98	0.92	0.93	0.92
	MACCS	0.96	0.96	0.96	0.88	0.89	0.88
	KRFP	0.95	0.95	0.95	0.89	0.89	0.89
RF	MolDesc	1.00	1.00	1.00	0.88	0.89	0.89
	ECFP	1.00	1.00	1.00	0.92	0.93	0.92
	MACCS	0.98	0.98	0.98	0.88	0.89	0.88
	KRFP	0.99	0.99	0.99	0.90	0.90	0.90
SVM	MolDesc	0.90	0.90	0.90	0.87	0.88	0.87
	ECFP	0.96	0.96	0.96	0.90	0.91	0.91
	MACCS	0.96	0.96	0.96	0.88	0.89	0.88
	KRFP	0.89	0.89	0.89	0.82	0.84	0.82
MLP	MolDesc	0.99	0.99	0.99	0.86	0.87	0.86
	ECFP	0.98	0.98	0.98	0.91	0.91	0.91
	MACCS	0.84	0.84	0.84	0.81	0.82	0.81
	KRFP	0.99	0.99	0.99	0.89	0.89	0.89
LR	MolDesc	0.84	0.84	0.84	0.83	0.84	0.83
	ECFP	0.98	0.98	0.98	0.91	0.92	0.91
	MACCS	0.74	0.75	0.74	0.73	0.75	0.73
	KRFP	0.90	0.90	0.90	0.86	0.87	0.86

^1^ MolDesc: molecular descriptors.

## Data Availability

Data are available at https://github.com/cptbern/5HT2A_ML and has been archived in Zenodo to ensure long-term accessibility and data integrity (https://doi.org/10.5281/zenodo.19493235).

## Supplementary Materials

The following supporting information can be downloaded at: https://www.mdpi.com/article/10.3390/molecules31111888/s1, Figure S1: Distribution of pKi values (negative logarithm of the Ki) of the whole data set, Figure S2: Confusion matrices for XGB on the test dataset, Figure S3: Confusion matrices for Random Forest on the test dataset, Figure S4: Confusion matrices for SVM on the test dataset, Figure S5: Confusion matrices for MLP on the test dataset, Figure S6: Confusion matrices for Logistic Regression on the test dataset, Table S1: Results y-randomization using XGBoost, Table S2: Explanation of molecular descriptors.

## Abbreviations

The following abbreviations are used in this manuscript:

5-HT_2A_	5-hydroxytryptamine receptor 2A
ALD-52	1-acetyl- lysergic acid diethylamide
BBB	Blood–brain barrier
CHO	Chinese hamster ovaries
CNS	Central nervous system
DMT	N,N-dimethyltryptamine
DOB	2,5-Dimethoxy-4-bromoamphetamine
[^125^I]DOI	1-(4-(125I)iodanyl-2,5-dimethoxyphenyl)propan-2-amine
ECFP	Extended connectivity fingerprints
GPCR	G protein-coupled receptor
HEK	Human embryonic kidney
Ki	Affinity
KRFP	Klekota Roth fingerprints
LR	Logistic regression
LSD	Lysergic acid diethylamide
MACCS	Molecular ACCess System
ML	Machine learning
MLP	Multi-layer perceptron
NPS	New psychoactive substances
pKi	Negative decimal logarithm of the affinity
QSAR	Quantitative structure–activity relationship
RF	Random Forest
SHAP	Shapley Additive exPlanations
SMILES	Simplified Molecular Input Line Entry System
SVM	Support Vector Machine
xAI	Explainable artificial intelligence
XGB	eXtreme gradient boosting
