# Supplementary material for: Explainable Artificial Intelligence (xAI) for 5-HT2A Receptor Binding Affinity of New Psychoactive Substances
Source: Molecules. 2026 Jun 1;31(11):1888. doi: 10.3390/molecules31111888 (PMC13257649; doi:10.3390/molecules31111888)
Supplement: Supplementary file 1 [file molecules-31-01888-s001.zip › molecules-4210408-supplementary.pdf]

Explainable Artificial Intelligence (xAI) for 5-HT_{2A} receptor binding affinity of New Psychoactive Substances

Authors

Verena Schöning, Katharina Elisabeth Grafinger, Daniel Pasin, Christophe P. Stove, Wolfgang Weinmann, and Felix Hammann

Figure S1:	Distribution of pKi values (negative logarithm of the Ki) of the whole data set.	2
Figure S2	Confusion matrices for XGB on the test dataset	2
Figure S3	Confusion matrices for Random Forest on the test dataset	3
Figure S4	Confusion matrices for SVM on the test dataset	4
Figure S5	Confusion matrices for MLP on the test dataset	5
Figure S6	Confusion matrices for Logistic Regression on the test dataset	6
Table S1:	Results y-randomization using XGBoost	6
Table S2:	Explanation of molecular descriptors	7

1 Figures

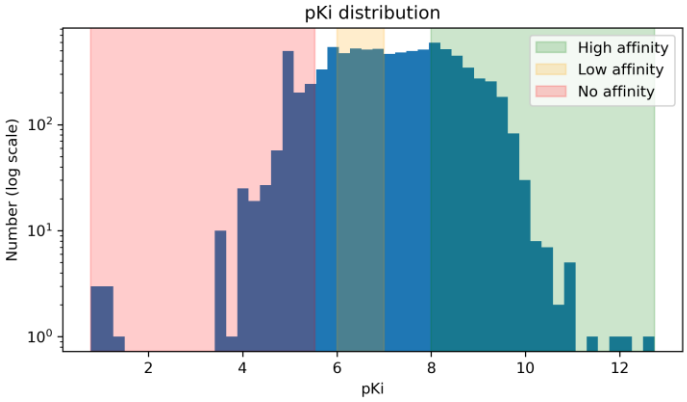

Figure S1 Distribution of pKi values (negative logarithm of the Ki) of the whole data set.

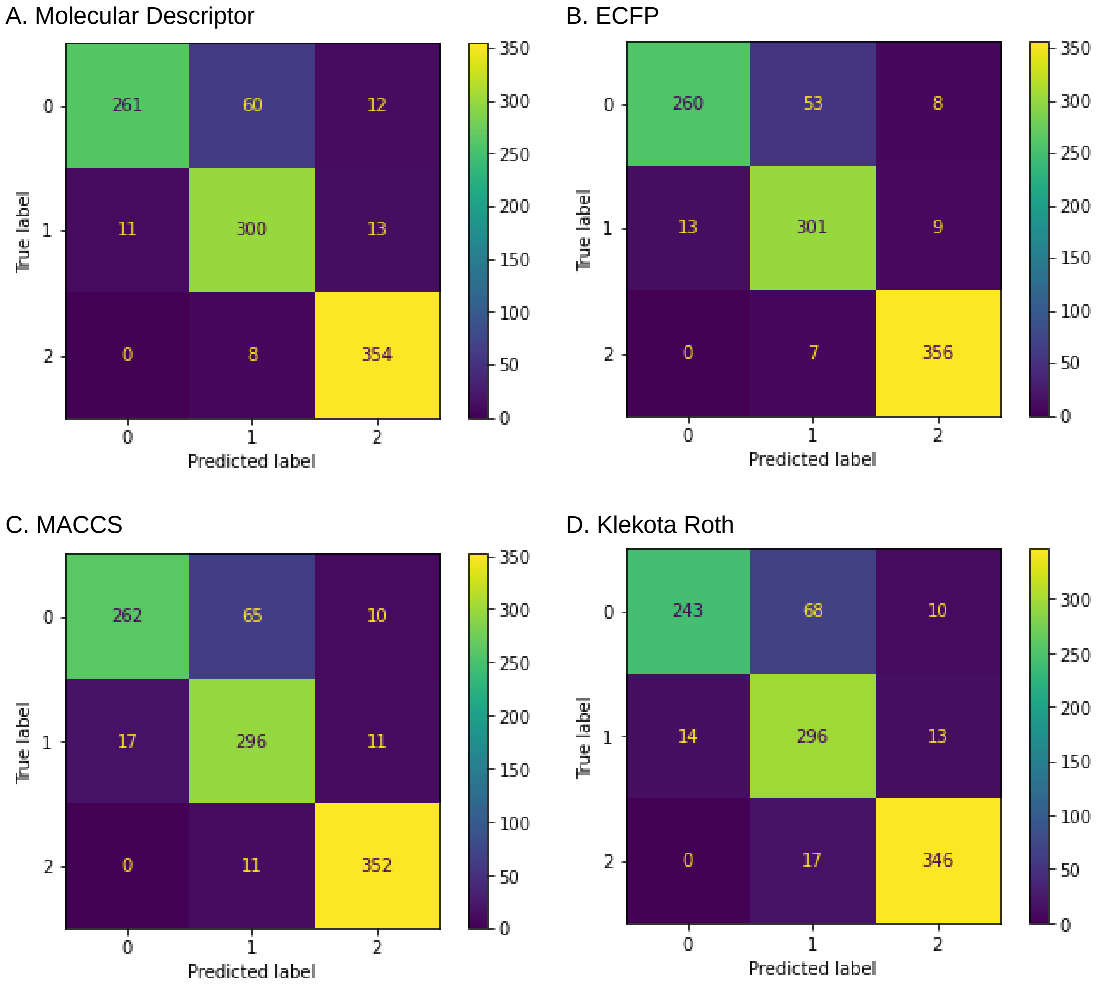

Figure S2 Confusion matrices for XGB on the test dataset

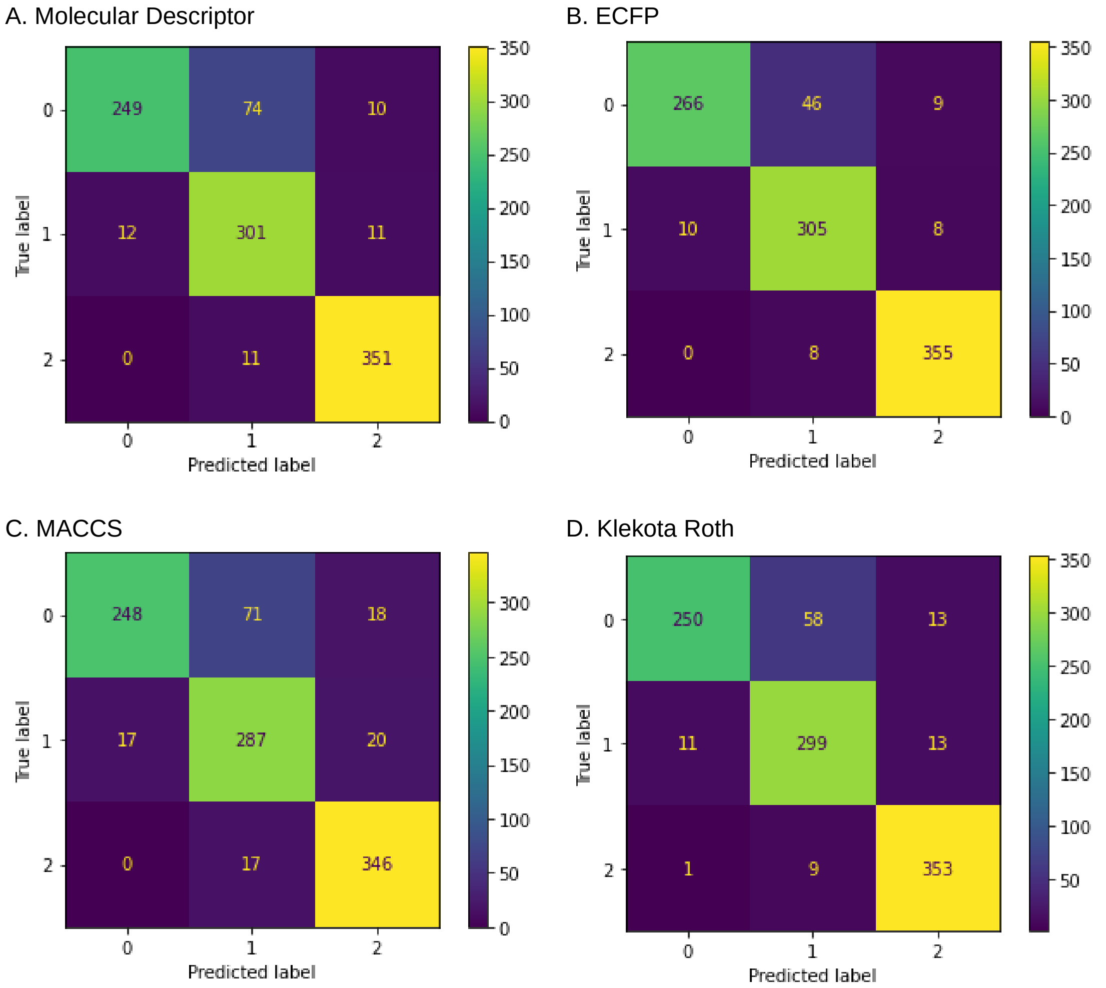

Figure S3 Confusion matrices for Random Forest on the test dataset

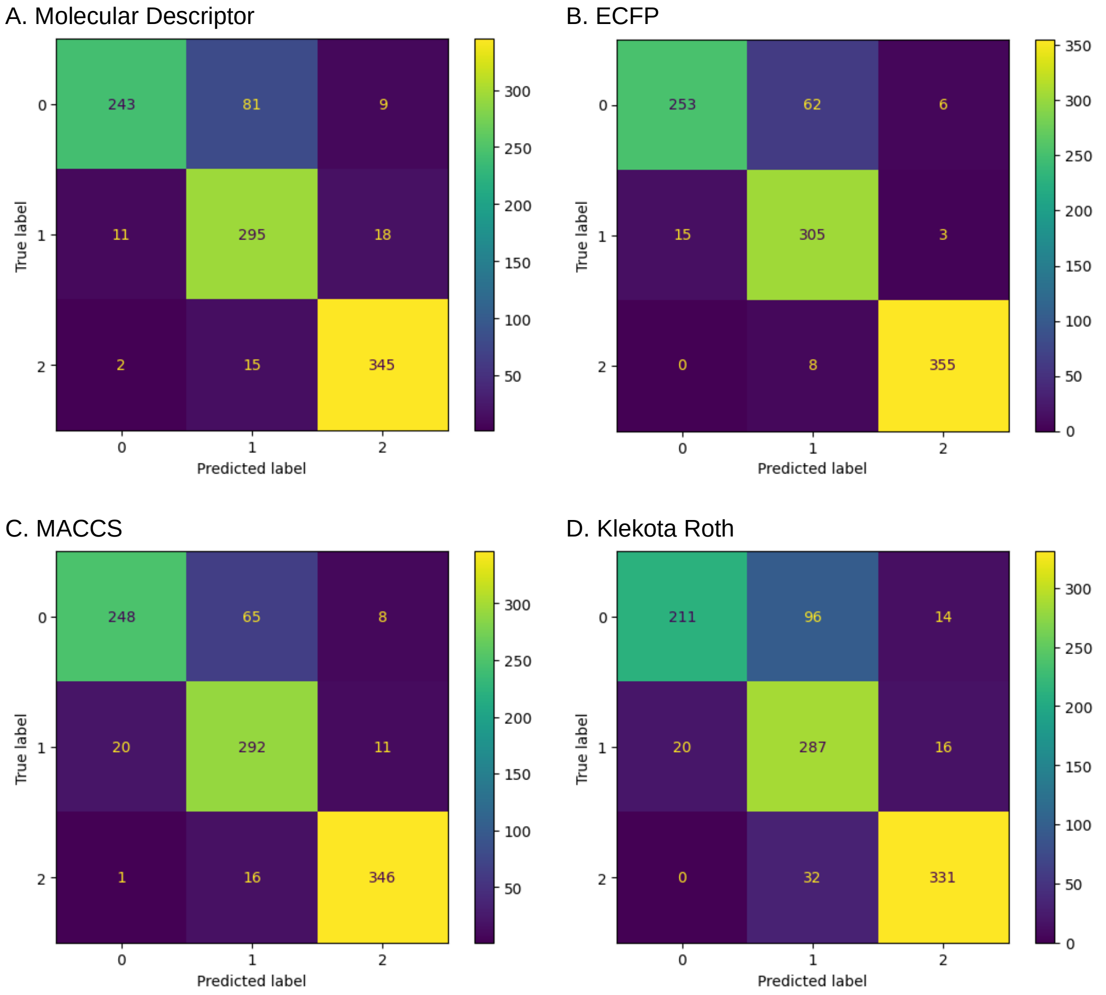

Figure S4 Confusion matrices for SVM on the test dataset

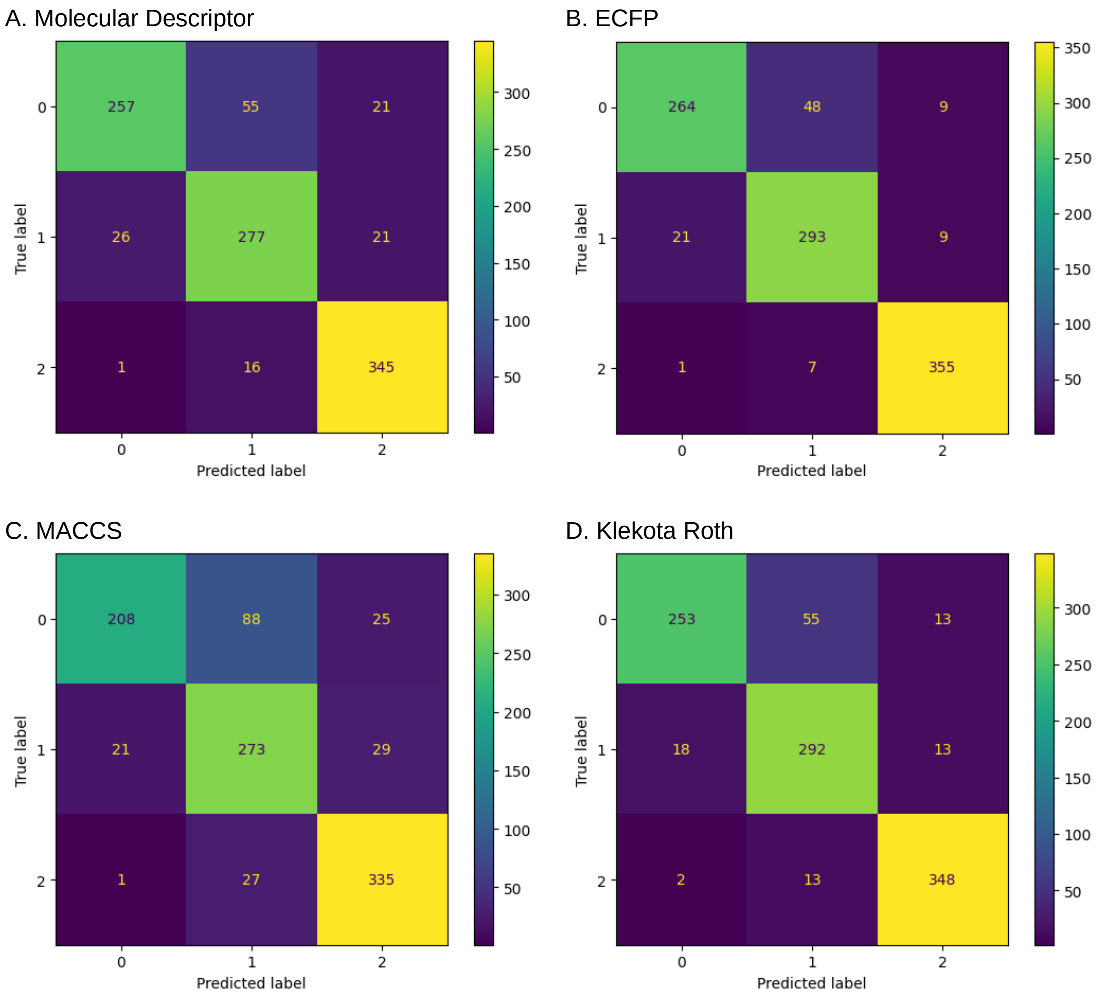

Figure S5 Confusion matrices for MLP on the test dataset

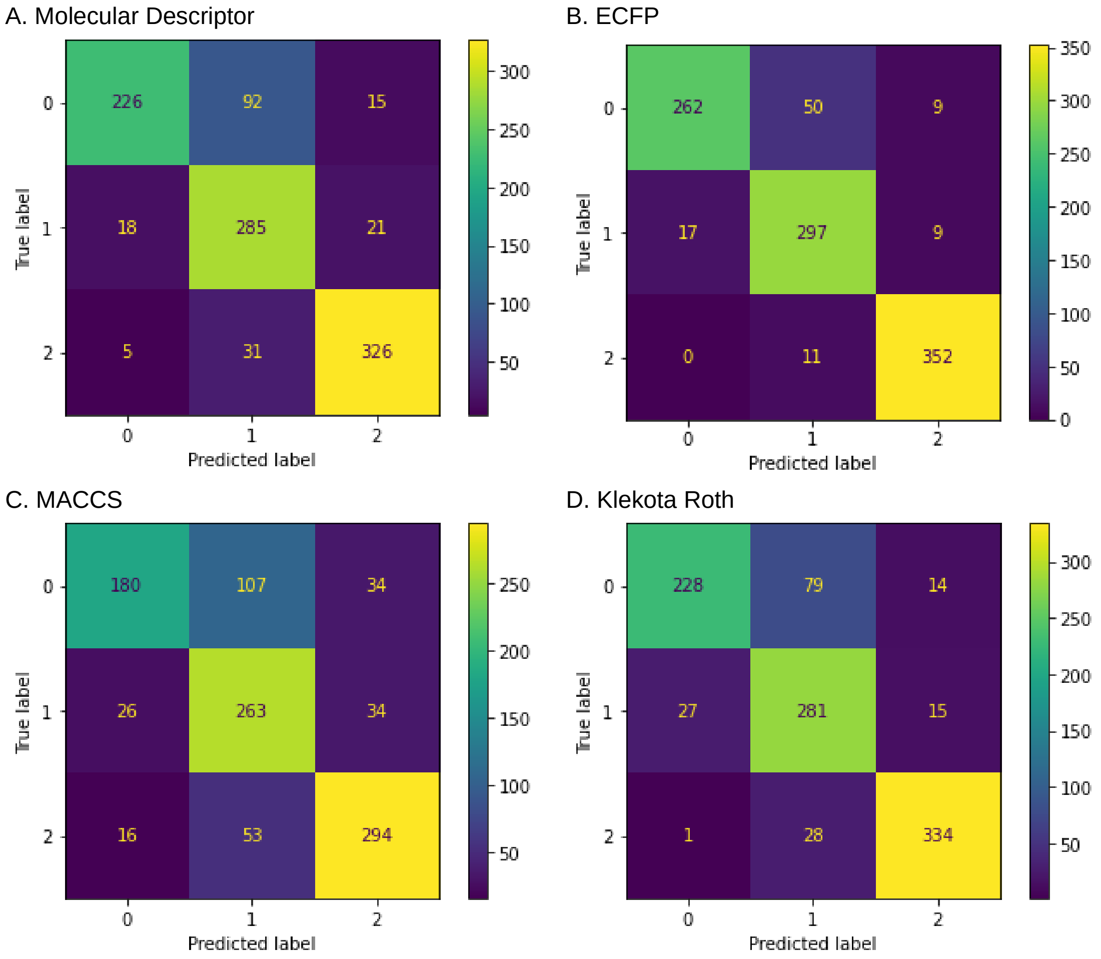

Figure S6 Confusion matrices for Logistic Regression on the test dataset

2 Tables

Table S1 Results y-randomization using XGBoost

	Training data set			Test data set		
**Descriptors**	**F1 score**	**Precision**	**Recall**	**F1 score**	**Precision**	**Recall**
MolDesc	0.98	0.98	0.98	0.45	0.46	0.46
ECFP	0.91	0.91	0.91	0.48	0.49	0.48
MACCS	0.82	0.82	0.82	0.45	0.45	0.45
Klekota Roth	0.70	0.71	0.71	0.43	0.43	0.43

MolDesc: Molecular Descriptor

Table S2 Explanation of molecular descriptors

Descriptor Class	Feature	Explanation
Atom type electrotopological state	LipoaffinityIndex	Lipoaffinity index
	maxaaCH	Maximum atom-type E-State: :CH:
	maxaasC	Maximum atom-type E-State: :C:-
	minaaaC	Minimum atom-type E-State: ::C:
	minHBa	Minimum E-States for (strong) Hydrogen Bond acceptors
	minHBint5	Minimum E-State descriptors of strength for potential Hydrogen Bonds of path length 5
	minsF	Minimum atom-type E-State: -F
	minssCH	Minimum atom-type E-State: -CH2-
	minssCH2	Minimum atom-type E-State: -CH2-
	SaasC	Sum of atom-type E-State: :C:-
	SssNH	Sum of atom-type E-State: -NH-
	SsssN	Sum of atom-type E-State: >N-
Autocorrelation	AATSC0i	Average centered Broto-Moreau autocorrelation - lag 0 / weighted by first ionization potential
	AATSC5c	Average centered Broto-Moreau autocorrelation - lag 5 / weighted by charges
	ATSC3e	Centered Broto-Moreau autocorrelation - lag 3 / weighted by Sanderson electronegativities
	ATSC6m	Average centered Broto-Moreau autocorrelation - lag 6 / weighted by mass
	GATS2c	Geary autocorrelation - lag 2 / weighted by charges
	GATS5i	Geary autocorrelation - lag 5 / weighted by first ionization potential
	MATS1c	Moran autocorrelation - lag 1 / weighted by charges
Barysz matrix	VE1_Dzp	Coefficient sum of the last eigenvector from Barysz matrix / weighted by polarizabilities
Burden modified eigenvalues	SpMax2_Bhs	Largest absolute eigenvalue of Burden modified matrix - n 2 / weighted by relative I-state
	SpMax3_Bhv	Largest absolute eigenvalue of Burden modified matrix - n 3 / weighted by relative van der Waals volumes
	SpMin1_Bhm	Smallest absolute eigenvalue of Burden modified matrix - n 1 / weighted by relative mass
	SpMin1_Bhv	Smallest absolute eigenvalue of Burden modified matrix - n 1 / weighted by relative van der Waals volumes
Information content	IC3	Information content index (neighborhood symmetry of 3-order)
Molecular distance edge	MDEC-23	Molecular distance edge between all secondary and tertiary carbons
Topological charge	GGI5	Topological charge index of order 5
	GGI7	Topological charge index of order 7
	JGI10	Mean topological charge index of order 10
	JGI3	Mean topological charge index of order 3
	JGI5	Mean topological charge index of order 5
XlogP	XlogP	XlogP
